# Physical fitness training in Subacute Stroke (PHYS-STROKE) - study protocol for a randomised controlled trial

**DOI:** 10.1186/1745-6215-15-45

**Published:** 2014-02-03

**Authors:** Agnes Flöel, Cordula Werner, Ulrike Grittner, Stefan Hesse, Michael Jöbges, Janet Knauss, Michael Seifert, Elisabeth Steinhagen-Thiessen, Mehmet Gövercin, Christian Dohle, Wolfgang Fischer, Regina Schlieder, Alexander Heinrich Nave, Andreas Meisel, Martin Ebinger, Ian Wellwood

**Affiliations:** 1 Center for Stroke Research Berlin, Charité - Universitätsmedizin Berlin, Berlin, Germany; 2 NeuroCure Clinical Research Center, Charité - Universitätsmedizin Berlin, Berlin, Germany; 3 Department of Neurology, Charité - Universitätsmedizin Berlin, Berlin, Germany; 4 Medical Park, Berlin, Germany; 5 Department for Biostatistics and Clinical Epidemiology, Charité - Universitätsmedizin Berlin, Berlin, Germany; 6 Brandenburg Klinik, Bernau, Germany; 7 Median Klinik, Grünheide, Germany; 8 Department of Geriatric Medicine, Charité - Universitätsmedizin Berlin, Berlin, Germany; 9 Median Klinik Berlin-Kladow, Berlin, Germany; 10 Center for Rehabilitation Research, University of Potsdam, Potsdam, Germany; 11 Beelitz-Heilstätten, Beelitz, Germany

## Abstract

**Background:**

Given the rising number of strokes worldwide, and the large number of individuals left with disabilities after stroke, novel strategies to reduce disability, increase functions in the motor and the cognitive domains, and improve quality of life are of major importance. Physical activity is a promising intervention to address these challenges but, as yet, there is no study demonstrating definite outcomes. Our objective is to assess whether additional treatment in the form of physical fitness-based training for patients early after stroke will provide benefits in terms of functional outcomes, in particular gait speed and the Barthel Index (co-primary outcome measures) reflecting activities of daily living (ADL). We will gather secondary functional outcomes as well as mechanistic parameters in an exploratory approach.

**Methods/Design:**

Our phase III randomised controlled trial will recruit 215 adults with moderate to severe limitations of walking and ADL 5 to 45 days after stroke onset. Participants will be stratified for the prognostic variables of “centre”, “age”, and “stroke severity”, and randomly assigned to one of two groups. The interventional group receives physical fitness training delivered as supported or unsupported treadmill training (cardiovascular active aerobic training; five times per week, over 4 weeks; each session 50 minutes; total of 20 additional physical fitness training sessions) in addition to standard rehabilitation treatment. The control intervention consists of relaxation sessions (non-cardiovascular active; five times per week week, over 4 weeks; each session 50 minutes) in addition to standard rehabilitation treatment. Co-primary efficacy endpoints will be gait speed (in m/s, 10 m walk) and the Barthel Index (100 points total) at 3 months post-stroke, compared to baseline measurements. Secondary outcomes include standard measures of quality of life, sleep and mood, cognition, arm function, maximal oxygen uptake, and cardiovascular risk factors including blood pressure, pulse, waist-to-hip ratio, markers of inflammation, immunity and the insulin-glucose pathway, lipid profile, and others.

**Discussion:**

The goal of this endpoint-blinded, phase III randomised controlled trial is to provide evidence to guide post-stroke physical fitness-based rehabilitation programmes, and to elucidate the mechanisms underlying this intervention.

**Trial registration:**

Registered in ClinicalTrials.gov with the Identifier NCT01953549.

## Background

### General

Stroke causes substantial personal costs in terms of disability and reduced social participation and exerts a considerable healthcare burden on informal care providers, healthcare providers and society in general. Two-thirds of stroke survivors suffer from residual neurological deficits and have to cope with chronic motor, language and cognitive dysfunctions [1]. Persistent motor deficits impair activities of daily living (ADL), such as dressing, self-care, and communicating [2]. Persisting cognitive and language deficits after stroke contribute significantly to permanent disability and emotional suffering in patients [3]. So far, we have limited evidence for the effectiveness of therapies in spite of intensive research efforts and numerous clinical trials [2].

Given the rising number of strokes worldwide [4], and the high prevalence of disabilities after stroke, novel strategies to reduce disability, increase functions (including communication and cognitive function), and improve quality of life are of major importance.

### Evidence for physical activity in post-stroke rehabilitation

First, animal studies have demonstrated that physical activity enhances neural plasticity and learning, processes central to functional recovery after stroke, by increasing neurotrophic substances like brain-derived neurotrophic factor, neurotransmitters such as dopamine, long-term potentiation, and possibly even neurogenesis [5].

Second, physical therapies are known to promote structural brain remodelling in humans [6], and this can influence post-stroke motor deficits. A recent systematic review indicated that repetitive practice of some common day-to-day activities lead to modest improvements in mobility and ADL in stroke patients [7].

However, conclusive evidence for beneficial effects of physical activity after stroke on ADL and walking speed is still missing. Most of the studies included in the 2011 meta-analysis by Brazzelli and colleagues [8] on physical activity-based interventions featured limited sample sizes and had a relatively short-term follow-up. Moreover, a variety of interventions were tested in diverse stroke populations with a range of outcome measures. The trials varied substantially in training intensity and frequency, total number of sessions, timing of training (acute, subacute or chronic), and locomotor impairment severity (non-ambulators to community ambulators), rendering it difficult to draw definite conclusions. Results indicate modest treatment effects associated with physical fitness training for stroke patients, particularly around interventions targeting “fitness” approaches rather than “strengthening”. Significant effects were demonstrated for an increase in maximal oxygen uptake and walking speed, with promising results for ADL (after intervention and on follow-up; both trends), and quality of life (directly after intervention). However, a subsequently published large trial on 408 stroke patients [9], comparing treadmill-based locomotor training either 2 or 6 months post-stroke to progressive exercise at home (non-specific, low intensity exercise intervention), did not find a significant benefit of locomotor training with regard to increasing the proportion of study participants who had higher functional walking levels (comfortable walking speed) at 1 year post-stroke. Of note, participants entered the trial only 2 months post-stroke in an ambulatory setting, and treadmill training was not aimed at enhancing cardiovascular fitness.

Third, in healthy humans undergoing physical fitness training, an increase not only in physical fitness, gait speed, and motor function but also in cognitive functions such as memory [10], executive functions [11], and language learning [12] have been demonstrated. Therefore, patients with not only motor deficits but also language and other cognitive dysfunctions may benefit from physical fitness training, a hypothesis that has not been addressed in most studies.

Fourth, profound effects on mood have been demonstrated by physical activity immediately after intervention in stroke [13]. Therefore, mood and quality of life measures post-stroke might be expected to improve at long-term follow-up, possibly depending on continuation of exercise.

Fifth, post-stroke physical activity and fitness levels are low, and these low levels are associated with common post-stroke functional limitations [14,15]. Increased fitness and physical function, shown to result from post-stroke physical activity training [8,14], could benefit a range of other common post-stroke problems by reducing fatigue, reducing the incidence of falls and fractures, compensating for the increased energy cost of a hemiparetic gait, reducing disability and improving independence [14,16].

Sixth, physical fitness training is beneficial for people with other comorbidities or risk factors for stroke. Systematic review evidence shows that interventions involving physical fitness training reduce blood pressure [17], improve vascular risk factors such as obesity [18] and type II diabetes [19], and reduce mortality in coronary heart disease. Therefore, post-stroke cardiorespiratory training, in particular, could reduce morbidity and mortality through secondary prevention of stroke and comorbid conditions.

In summary, physical fitness training, delivered as cardiovascular active, supported or unsupported ambulation training, may benefit a range of common post-stroke problems. It is likely that it does not simply provide a mechanism to increase fitness and improve walking ability, but has multiple mechanisms of action such as enhancing neurotrophins and overall plasticity and thus possibly exerts neuroplastic effects on motor, language and cognitive systems [5]. Therefore, it provides a spectrum of plausible benefits that are relevant to many people with stroke. However, there may also be risks, such as training-induced vascular events, soft tissue injuries, fatigue, altered muscle tone, and falls [20], although there is good evidence that cardiovascular active training can be administered successfully even in severely affected stroke patients [8,21,22].

### The need for a trial on physical activity after stroke

Physical activity may be a promising intervention to address post-stroke functional challenges but as yet there is no definitive study and a lack of data that can be widely applied. With regard to previous trials [8,9], several key questions remain to be answered. Firstly, a physical fitness intervention starting in the early rehabilitative phase after stroke, in an inpatient rehabilitation hospital setting with both ambulatory and non-ambulatory stroke patients has not been systematically assessed in a large number of patients. Secondly, the range of possible benefits including not only gait speed and functional ambulation category (FAC) but also ADL, arm function, quality of life and language as well as cognitive functions both immediately after intervention and on follow-up remains to be fully explored. Thirdly, an optimal physical fitness intervention prescription for people early after stroke has yet to be defined - for example, an intervention that accomplishes actual active cardiovascular training. This is an important goal: an increase in aerobic capacity may not only improve long-term cardiovascular health outcomes, but also promote functional abilities and independent living (for review see [14,15]). Contemporary stroke rehabilitation programmes [23] are generally far too low in intensity to induce a positive training effect [16].

### Objective

The objective of this study is to assess whether additional treatment in the form of physical fitness-based training, delivered as supported or unsupported treadmill or electromechanical gait training, for patients early after stroke will provide benefits in terms of outcomes that reflect their daily living - in particular gait speed and the Barthel Index (BI), a measure of ADL (co-primary outcome measures), both immediately after the intervention, 3 months (after stroke onset) (primary outcome), and at medium-term (6 months) follow-up. Using an exploratory approach we will also gather data on arm function, quality of life, sleep and mood, cognitive scores, and maximal oxygen uptake.

## Methods/Design

### Type of design

**Figure 1:**
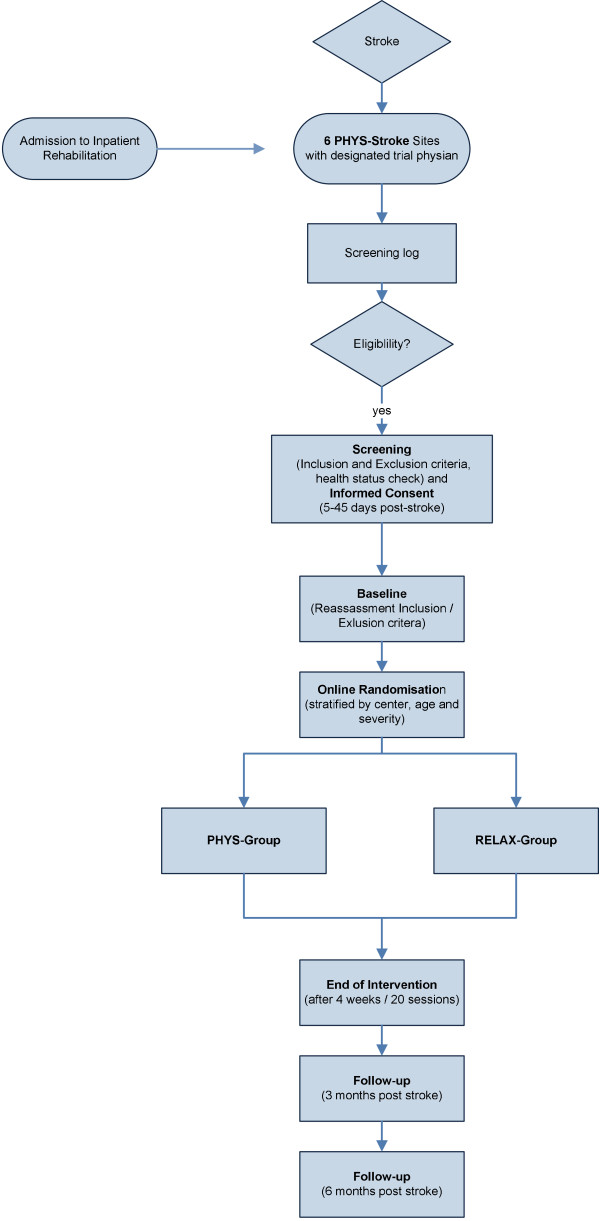
**Flow diagram of the Physical fitness training in Subacute Stroke (PHYS-STROKE) study design.** PHYS-Group, physical activity experimental intervention group; RELAX-Group, relaxation active control intervention group.

This is a multi-site, two-arm, endpoint-blinded, phase III randomised controlled trial comparing 4 weeks physical fitness training in addition to standard rehabilitation treatment (experimental intervention) versus 4 weeks relaxation treatment in addition to standard rehabilitation treatment (active control intervention). The primary outcomes will be gait speed (in m/s, 10 m walk) and ADL (as measured by the BI) 3 months after stroke (co-primary endpoints). For study design, see Figure 1.

### Study enrolment

Individuals post-stroke aged 18 years or older are recruited 5 to 45 days post-stroke at six clinical sites (inpatient rehabilitation hospitals in or around Berlin).

### Screening process

All patients entering the six rehabilitation hospitals with the primary diagnosis of stroke [24] will be entered into a screening log by the designated trial physician of the respective site. Subsequently, the patient will be screened for eligibility with the inclusion and exclusion criteria as outlined below (see also Table 1). Eligible patients will be fully informed of the study, and a patient screening identification number will be assigned to the patient. Patients are then asked to provide informed consent (see below). The screening process allows us to determine the number of eligible patients. In the event that a potential participant does not meet the inclusion criteria, no identifiable information is retained on that participant. Reasons for exclusion will be reported.

**Table 1:** **Inclusion and exclusion criteria of Physical fitness training in Subacute Stroke** (**PHYS-STROKE) study**

**Inclusion criteria**	
1.	Diagnosis of stroke (within 5–45 days after stroke); ischaemic or haemorrhagic (cortical, subcortical, brainstem), as determined by initial MRI/CT scan of the brain)
2.	Age ≥18 years
3.	Able to sit for at least 30 seconds (unsupported or supported - that is, holding onto supports such as the edge of the bed)
4.	Barthel Index ≤65 at inclusion
5.	Considered able to perform aerobic exercise, as determined by responsible physician
6.	Provision of written informed consent
**Exclusion criteria**	
1.	Patient considered unable to comply with study requirements
2.	Stroke due to intracranial haemorrhage primarily due to bleeding from ruptured aneurysm or arteriovenous malformation
3.	Progressive stroke
4.	Unable to perform the required exercises due to a) medical, b) musculo-skeletal, or c) neurological problems (for details see below, 4a-c)
4a.	medical problems: unstable cardiovascular condition, or other serious cardiac conditions (for example, anyone meeting New York Heart Association Class IV criteria, hospitalisation for myocardial infarction or heart surgery within 120 days, severe cardiomyopathy or documented serious and unstable cardiac arrhythmias)
4b.	musculo-skeletal problems: restricted passive range of motion in the major lower limb joints (that is, an extension deficit of >20° for the affected hip or knee joints, or a dorsiflexion deficit of >20° for the affected ankle)
4c.	neurological problems: severity of stroke-related deficits
5.	Required help of at least 1 person to walk before stroke due to neurological (for example, advanced Parkinson’s disease, amyotrophic lateral sclerosis, multiple sclerosis) or non-neurological (for example, heart failure, orthopaedic problems) co-morbidities
6.	With life expectancy of less than 1 year as determined by responsible physician
7.	Drug or alcohol addiction within the last 6 months
8.	Significant current psychiatric illness defined as affective disorder unresponsive to medication or bipolar affective disorder, psychosis, schizophrenia or suicidality
9.	Current participation in another interventional trial

CT, computer tomography; MRI, magnetic resonance imaging.

Subsequently, the designated trial physician will record all baseline medical and neurological information in the case report form. One of the designated study assessors will be assigned to the patient, and conduct all baseline assessments (see “Study outcome assessment” and Table 2) at the respective rehabilitation centre. The study assessor is part of the study team and will not be involved with treatment of patients or with administration of the intervention, and will remain fully blinded to the patient’s group allocation throughout the trial.

**Table 2:** Study outcome assessment

	**Baseline**	**End of intervention**	**Follow-up (3 months)**	**Follow-up (6 months)**
**Primary outcome (co-primary)**				
Gait speed (in m/s, 10 m walk)	X		X	
Barthel Index	X		X	
**Secondary outcome**				
Gait speed (in m/s, 10 m walk)		X		X
Barthel Index		X		X
**Mobility**				
Gait endurance (6-minute walk)	X	X	X	X
Actigraph	X	X	X	X
Rivermead mobility index	X	X	X	X
**Motor function**				
Rivermead arm test	X	X	X	X
Box and block test	X	X	X	X
REPAS scale	X	X	X	X
Medical research council scale	X	X	X	X
Functional ambulation classification	X	X	X	X
**Cognitive function**				
Montreal cognitive assessment	X	X	X	X
Trail Making test A and B	X	X	X	X
Semantic and phonemic word fluency	X	X	X	X
**Disability, quality of life, sleep, mood**				
Modified rankin scale	X	X	X	X
EQ-5D-5 L	X	X	X	X
Pittsburgh sleep quality index	X	X	X	X
CES-D scale	X	X	X	X
**Physical fitness**				
Maximal oxygen uptake and gait energy expenditure	X	X	X	X
**Cardiovascular risk factors, others**				
Systolic/diastolic blood pressure, heart rate	X	X	X	X
Weight	X			
Waist-to-hip-ratio	X	X	X	X
Laboratory tests (blood draw)	X	X	X	X
Hair cortisol concentration (strand of hair)	X			

CES-D Scale, Center for Epidemiological Studies Depression Scale; EQ-5D-5 L, Euro Quality of Life 5 Dimension 5 Level scale; REPAS scale, REsistance to PASsive movement Scale.

### Informed consent

Written informed consent for study participation is obtained at 5 to 45 days post-stroke (see above). Participants are informed that successful completion of the baseline tests is required prior to randomisation to an intervention group.

### Inclusion and exclusion criteria

Participants are individuals with recent onset of ischaemic or haemorrhagic stroke. For purposes of inclusion in this study, a stroke is defined according to the World Health Organization definition as “a rapid onset event of vascular origin reflecting a focal disturbance of cerebral function, excluding isolated impairments of higher function and persisting longer than 24 hours” [24]. Stroke diagnosis is confirmed by computed tomography or magnetic resonance imaging scan (for details see Table 1).

### Study outcome assessments

#### Methods and timing of assessments

Measures selected have established reliability and validity and are performed according to standardised protocols defined in an operations manual. Blinded study assessors conduct all baseline and follow-up assessments (after 4 weeks intervention, 3 and 6 months post-stroke). Participants are instructed after randomisation to refrain from discussing assignment group during evaluations. In addition, a reminder card is handed to each patient at the beginning of each evaluation session to remind them not to reveal their group assignment. To determine the effectiveness of our blinding strategy, the study assessor will be asked if they were or were not “unblinded”. In addition, during the intervention all additional therapy sessions are recorded (for details, see “Additional information” below).

Cerebral blood flow and volume, functional and structural networks (based on magnetic resonance imaging) as well as blood biomarkers of endothelial function will be determined in a subgroup of patients at baseline and at the end of the intervention (for details see ClinicalTrials.gov with the Identifier NCT01954797).

### Measures

#### Primary efficacy endpoints (co-primary endpoints)

Co-primary endpoints are change in gait speed (in m/s, 10 m walk) and BI (100 points total) at 3 months post-stroke compared to baseline measurements. Co-primary endpoints are chosen so that a comprehensive picture of the treatment effect can be obtained [25]. Because each primary endpoint can characterise a clinically meaningful benefit of the intervention on its own, we use the “or decision rule”, meaning that the study is regarded as successful in case that one of the two primary endpoints improves significantly in the intervention group.

BI is chosen as one of the primary outcome measures since it assesses disability, and improving disability is a primary goal of physical therapy interventions post-stroke. BI is largely based on mobility*,* which will be targeted by treadmill-based physical fitness training. Additionally, BI is widely used in the rehabilitative setting in Germany and is the primary parameter for allocation to rehabilitation facilities by healthcare insurers. A relatively large trial [22] demonstrated that treadmill training may significantly enhance BI scores in a cohort of severely affected patients in a subacute inpatient rehabilitation setting. We acknowledge concerns about the ceiling effect of BI but, given the severity of disability at baseline in our patient population, we consider the risk of it masking a positive effect of the intervention to be minimal and acceptable. The inclusion criteria specify “BI at baseline ≤65 points”. A difference of 10 points of the BI is considered a clinically meaningful difference [26].

Gait speed is chosen because it is related to the severity of impairment in the home and the community, and functional walking capacity is a primary goal of many physical therapy interventions post-stroke. Moreover, previous studies in post-stroke patients that involved treadmill-based physical fitness training have most often used gait-related primary outcome measures [8,9]. Gait speed (m/s) will be assessed with the 10-m test: the patients will walk a distance of 14 m (2 m for acceleration and deceleration) at their maximum speed twice. The time will be taken and the mean speed (m/s) calculated [22,27,28]. A difference of 0.1 m/s on short distance walks (for example, ambulation around the house) is considered a clinically meaningful difference [29,30].

#### Key secondary endpoints

Change in gait speed and BI at the end of intervention and at 6 months post-stroke, compared to baseline gait speed and BI, will constitute secondary endpoints.

Additional secondary endpoints, to be assessed at baseline, end of intervention, and 3 and 6 months post-stroke include the following (see also Table 2 and Additional file 1): mobility (gait endurance, Actigraph, Rivermead Mobility Index), motor function and spasticity (Rivermead Arm Test, Box and Block Test, Medical Research Council scale, REsistance to PASsive movement scale), cognition (Montreal Cognitive Assessment, Trail Making Test A and B, Semantic and Phonemic Word Fluency), disability, mood and quality of life (Euro Quality of Life 5 Dimension 5 Level scale, Pittsburgh Sleep Quality Index, Center for Epidemiological Studies Depression scale, modified Rankin scale), and physical fitness (maximal oxygen uptake, gait energy expenditure).

Moreover, we will assess total time spent in physical activity per day (usual care intervention log; by physiotherapist), FAC, vascular risk factors (resting systolic and diastolic blood pressure; resting heart rate; body mass index, waist-to-hip ratio, markers of inflammation, immunity and the insulin-glucose pathway, lipid profile, and other laboratory parameters, derived by a blood draw from a peripheral vein, hair cortisol concentration by taking a small strand of hair [31]), length of time in rehabilitation, and medication.

For more details, see Additional file 1.

#### Assessment of safety

At each assessment (baseline, end of intervention, and 3 and 6 months post-stroke), the following parameters will be systematically recorded: recurrent fatal or non-fatal cardiovascular or cerebrovascular events; referral to an acute hospital; death.

After each intervention, the physiotherapist will record the presence of self-reported pain, fatigue, dizziness, number and nature of falls, and note other adverse events.

#### Additional information

Total time spent in rehabilitative therapies will be recorded by usual care intervention logs to track the amount of physical, occupational, speech and language, and cognitive therapy that Physical fitness training in Subacute Stroke (PHYS-STROKE) participants receive during enrolment in the trial as part of the usual care treatment. Therapists (during inpatient treatment, both during the trial intervention and beyond) and patients (in the months following inpatient rehabilitation) are instructed to write in the time (in minutes) of additional therapy they receive outside of their participation in the trial on monthly calendars provided for them. The patients will return the monthly calendar to the study assessor during follow-up visits.

Information on the medications taken by a participant is recorded at baseline and at the follow-up assessments. Length of time (in days) spent in rehabilitation will also be recorded.

### Standardisation of assessments

Standardisation of data collection methods is achieved through a systematic training and competency assessment programme for the blinded assessors. The assessors are informed of the battery of outcome measures in a lecture and demonstration. This is followed by practice on volunteers (usually persons with stroke) under the supervision of the study’s clinical research coordinators and site team leaders, and successful completion of competency training confirmed by a competency check-list.

### Randomisation

Each participant is assigned to one of two groups, either the physical activity (PHYS) group or the relaxation (RELAX) group by block-randomisation with stratification for centre, age (≤65 years, >65 years), and severity (functional ambulation category, FAC (0–3, 4–5)). Age and severity might influence functional outcome, and will therefore be included as stratification factors. Moreover, given the pragmatic approach for the application of physical fitness training, and given that different physiotherapists will administer the training in each rehabilitation site, the study centre will be another stratification factor.

The allocation of patients to the two groups (PHYS or RELAX group) will be conducted online by using the web-based randomisation tool of the Institute of Medical Informatics, statistics and documentation, Medical University of Graz, Austria; available at http://www.randomizer.at.

### Interventions

Participants are randomised to one of two intervention groups, each receiving five treatment sessions per week for 4 weeks (total of 20 sessions): 1) PHYS (physical activity group; target intervention); or 2) RELAX (relaxation group; control intervention).

Both target and control groups will receive any prescribed usual and customary rehabilitative care during the 4 weeks in addition to the PHYS and RELAX interventions. Customary rehabilitative care may involve cardiovascular active training. However, little cardiovascular active training is actually administered during the course of the normal rehabilitation programme (see our on-site pilot study; see also [16] for review). The fitness training in the target group will always be in addition to the normal rehabilitation programme. The reason to allow individuals to receive usual and customary rehabilitative care is that participants may be reluctant to enrol if they believe trial participation will reduce their opportunities to participate in other therapy. Usual care interventions are monitored by usual care intervention logs.

#### PHYS training programme

Physical fitness training (cardiovascular active; 50 to 60% of maximum heart rate) [32] will be delivered as supported or unsupported treadmill training or on an electromechanical gait trainer. To be cardiovascular active (that is, reaching training intensities of 50 to 60% of maximum heart rate; 180 beats per minute, Gordon and colleagues [21]), target heart rate will be determined according to the following formula: 180 – age. In case of a beta blocker medication, 10 beats will be subtracted; that is 180 – age – 10 = target heart rate (pragmatic approach; see [21,33]).

A graded increase of belt speed, reduction of body weight support and/or inclination will be used to elicit adequate cardiovascular stress to induce an aerobic training effect. During aerobic training, patients will wear a modified parachute harness to prevent falls. The body weight will be either unsupported or supported to a maximum of 15% of body weight according to individual needs. If necessary, one or two therapists will provide help with setting the paretic limb or assisting weight-shifting, and hip and knee extension.

Each session will start with a warm-up of 3 minutes, followed by 20 minutes exercise at target heart rate, and 2 minutes cool-down. If the patient indicates that they need to rest, or the therapist decides that rest is needed (heart rate; overall impression), a maximal 2-minute rest period (sitting) will be allowed, followed by a return to training heart rate over 1 to 2 minutes. Goals for each session address achieving cardiovascular active training duration of 20 minutes, and maximal body weight load while maintaining the kinematics and posture associated with walking. An early training priority is to achieve an upright symmetrical posture with spatial-temporal symmetry of the stepping pattern. Initially, the participant may walk with a shorter step length for the non-paretic limb and the step centred in the middle of the treadmill as a means to compensate for deficits in paretic limb and trunk control. A trainer thus initially works with the participant to verbally cue or manually assist with foot placement of the non-paretic limb.

#### RELAX training programme

Relaxation sessions will avoid cardiovascular active activity and aim for <30% of maximum heart rate [32]. Relaxation sessions will involve the relaxing of different muscle groups over the face, head, shoulders, arms, legs, chest, back, and abdomen, guided by the therapist and compact disc. With eyes closed and in a sequential pattern, the patient is encouraged to concentrate on the sensation of relaxation such as feelings of warmth and heaviness. This progressive training helps the participant achieve physical and mental relaxation and calmness [34], and does not convey any cardiovascular active training, but the same amount of additional contact by the therapist. Cardiovascular response monitoring during the RELAX intervention is identical to that done in the PHYS group. In this way, the RELAX intervention will plausibly control for the Hawthorne effect, but exclusively through interventions that have been shown to have little or no impact on aerobic exercise capacity. Each participant is individually progressed according to their ability within each phase..

#### Vital signs monitoring for PHYS and RELAX interventions

Blood pressure, heart rate and Borg scale Rate of Perceived Exertion will be monitored prior to a session and at the completion of each session. Heart rate will be monitored during each session. Heart rate must be less than 100 beats per minute to begin the training session. Resting diastolic blood pressure must be <100 mmHg and systolic blood pressure <180 mmHg to begin the training session [35]. The American College of Sports Medicine criteria for terminating an inpatient exercise session are followed according to guidelines shown to be effective for persons post-stroke with multiple comorbidities [35]. If the patient complains of angina at rest, loss of consciousness occurs, or cardiac arrest, emergency medical services are called immediately. All trainers are cardiopulmonary resuscitation certified and aware of signs of cardiac complications.

#### Standardisation for the interventions

The PHYS and RELAX interventions are standardised to achieve consistent implementation of the intervention across rehabilitation sites. Standardisation assures that the training teams successfully implement a common intervention through application of six critical elements: 1) knowledge of the protocol; 2) goal setting, decision-making and progression; 3) participant safety and monitoring; 4) equipment use; 5) hands-on training skills; and 6) participant’s role and participation.

Documentation procedures are standardised across sites and require trainers to record in a computerised database all training parameters. A competency-based training programme will be used to train the trainers across all sites. The intervention teams will meet for a 2-day training course, return to their clinical sites for 1 month of pilot training, with follow-up sessions at each of the six sites to complete competency-based training and testing. Competency is required in each of the six knowledge and application domains and includes both written and practical components.

After individual therapists and the site achieve competency status, they are approved to train patients in the randomised controlled trial. Competency status is maintained throughout the trial by each site and is regularly reviewed by the study team. Turnover in therapists across sites is anticipated across a 2.5 year span of participant entry and training. The intervention site team leader is responsible for training new staff. The therapist must achieve intervention competency before joining the site team in treating patients. Any deviation from established competency standards requires immediate retraining and re-evaluation. No therapist is allowed to conduct treatment without established and maintained competency.

The Clinical Research Coordinator and co-primary investigators are responsible for maintaining standardisation and competency throughout the trial. Communication between clinical research coordinators, co-primary investigators and centre representatives is maintained through weekly conference calls. A web-based discussion board specific to each intervention provides timely responses to questions from the training teams with responses available to all training personnel. This list of questions and responses is recorded throughout the trial and used to refine or clarify the training manual. The Clinical Research Coordinators conduct weekly to bi-monthly visits to each site and relay any intervention-related concerns to the primary investigators. Finally, the investigators have prepared an intervention training manual for therapists available at each clinical site.

### Statistical analysis

The following power calculation estimates the number of participants required for given primary outcomes and estimates of power. PHYS-STROKE is powered to detect a difference in gait speed of 0.13 m/s, and a difference in Barthel Index of 10 points between the two groups. A total of 172 patients (86 patients per group) will enter this study. Because of the analysis of two primary outcomes, the significance level was Bonferroni-corrected (α = 0.05/2 = 0.025).

*First co-primary endpoint: change in BI at 3 months follow-up compared to baseline.* We took a German “repetitive locomotor therapy” study (20 minutes on gait trainer + 25 minutes physiotherapy per day five times per week for 4 weeks) [22] as the basis for the power calculation for the outcomes measured on the BI. A difference of 10 points on the BI is considered a clinically meaningful difference [26]. With a power of 80%, PHYS-STROKE will detect a group difference at a two-sided 0.025 significance level, if the mean difference of improvement on the BI between groups is 10 points if 86 patients in each group will be included. This is based on the assumption that the common standard deviation of the response variable is 21 (at 3 month follow-up).

*Second co-primary endpoint: change in gait speed at 3 months follow-up.* A difference of 0.1 m/s on short distance walks is considered a clinically meaningful difference [29,30]. A sample size of 86 in each group will have 87% power to detect a difference between groups in means of improvement in gait speed of 0.13 m/s (mean improvement in intervention group of 0.31 m/s and mean improvement in control group 0.18 m/s [22]), assuming that the common standard deviation is 0.25 using a two group t-test with a 0.025 two-sided significance level.

Drop out was estimated at 25%, resulting in a trial with a total of 215 subjects. This is considered feasible for the six large recruiting centres over a period of 2.5 years recruitment.

Group differences for the co-primary endpoints will be analysed using analysis of covariance (ANCOVA) with baseline measures as covariates.

Here, the t-test was used for sample size estimation in spite of the intended analysis with an ANCOVA model. It can be shown [36] that this is a conservative approach for estimating samples sizes for ANCOVAS, because an ANCOVA with (1 – p^2^)*n subjects has the same power as a t-test with n subjects where p is the variance deflation factor, calculated by the correlation of baseline and follow-up measures. Assuming the worst case of p = 0 leads to the sample size based on the t-test.

#### Analysis plan

The analyses of primary and secondary endpoints will be performed in the intention-to-treat population consisting of all randomised patients who received at least 1 day of training. In patients lost to follow-up, their last observation will be carried forward in order to accomplish the intention-to-treat analysis.

The primary analysis will apply an ANCOVA with the two primary outcomes at follow-up as dependent variables, and baseline scores and group as independent variables. Additionally, the analysis will be adjusted for centre, age and severity (as assessed by the FAC) of stroke.

As a worst case sensitivity analysis, the primary analysis will be repeated by imputing baseline values for missing values instead of last observation carried forward. Regarding the secondary endpoints, results from the study will be analysed using standard statistical methods. For every endpoint it has to be checked in advance which covariates should be accounted for, and this will not be presented in detail here. Limitations resulting from the study design, the sample size or unmeasured confounders will be discussed. No adjustment for multiplicity of testing is provided. No interim analysis (other than a blinded sample size reassessment) is planned since the follow-up time is long as compared to the anticipated recruitment time.

A fully specified statistical analysis plan will be written before unblinding of the study. The final report of the trial will follow the CONSORT extension for non-drug trials.

### Avoidance of missing data

To limit missing data as much as possible, we have implemented the following steps. Participating sites and their primary investigators have been chosen from within the framework of the Berlin Stroke Alliance, an alliance of stroke care providers and researchers that has been set up in Berlin and Brandenburg and will allow us to recruit, treat and perform long-term follow-up on a sufficiently high number of stroke patients in a high-quality neurorehabilitation trial. All participating sites have worked together for several years, for example, to develop a core data set of the Berlin Stroke Alliance [37]. All sites participated in the set-up of the trial during several meetings over the last year, in which the participation of physiotherapy and nursing staff was ensured. Agreements between the central study centre and the sites with regard to basic trial equipment and case payments (considering missing data and drop-outs) have been concluded. Moreover, a questionnaire was sent to each site to evaluate the annual number of chronic stroke patients potentially available for the trial in that site, the clinical spectrum of patients, available experience with clinical trials and other currently competing studies.

Investigators and study staff are trained on the importance of keeping participants in the trial until the end, regardless of whether they continue to receive the assigned treatment, and this information is also conveyed to study participants. Even if a patient wishes to discontinue study treatment, all reasonable efforts will be made to obtain the patients’ informed consent for the collection of at least primary outcome data. Contact information for participants is kept up to date in order to prevent loss to follow-up. Moreover, physiotherapists delivering the intervention (target and control) are trained in providing a positive experience, and the amount of data collected by the trial assessors has been restricted as much as possible. Both patients and their relatives/care-givers will be provided with detailed and updated information on the background and purpose of the trial, to keep motivation to continue with the intervention and follow-ups as high as possible.

The on-site monitor will pay particular attention to missing data during the process of source data verification and the study centre will provide with further training if necessary. Furthermore, regular communication with the study community (for example, by study newsletter) is planned by the central study centre to ensure the profile and interest in the study is maintained.

### Handling of drop-outs/missing data

For patients that are not willing or able to complete their intervention (at least 75% of the intended training/relaxation sessions) but do not retract their informed consent, the co-primary endpoints gait speed and BI will be assessed after their last intervention, and at 3 months follow-up. Using the 3-month follow-up values, an intention-to-treat analysis will be possible. For those patients who drop out and are not willing to be assessed regarding gait speed and BI after 3 months, we will use adjusted regression-based multiple imputation methods using the information of the other patients to impute missing values and we will analyse both the full data set with imputed values and the smaller data set without imputed values in an intention-to-treat analysis [38,39].

### Adverse event monitoring and reporting

Adverse events are carefully monitored at every level of the PHYS-STROKE trial. A Data Safety Monitoring Board provides oversight and meets after randomisation of every 50 new patients. All adverse events are reported immediately to the responsible physician and the board members are informed of all serious adverse events.

For more details on data management, quality control and study organisation, see Additional file 1, sections entitled “Data Management and Quality Control Procedures” and “Study Organisation and Management”.

### Ethics

All procedures conducted during this trial will be carried out in compliance with institutional ethical standards and in compliance with the Declaration of Helsinki. All research procedures were approved by the responsible Institutional Review Boards (for more details, see Additional file 2).

### Dissemination policy

Trial results will be published first in scientific journals. Moreover, results will be made available for a scientific and a lay audience on the ClinicalTrials.gov website (Registered in ClinicalTrials.gov with the Identifier NCT01953549) [40]. As part of the Cochrane Collaboration Initiative, full access will be granted to the protocol, participant-level data set and statistical code, for future reviews for published data and individual patient data meta-analysis. Authorship will follow the “Uniform Requirements for Manuscripts Submitted to Biomedical Journals: Ethical Considerations in the Conduct and Reporting of Research: Authorship and Contributorship”, for details see http://www.icmje.org/ethical_1author.html. No professional writers will be employed.

## Discussion

This multi-site, randomised controlled trial is designed to determine the impact of cardiovascular active early physical fitness intervention, compared to a relaxation control condition, on clinically and functionally meaningful changes in ability and function for patients after stroke.

Considering the lack of knowledge with regard to evidence-based care, and the high level of medical interest in the area of rehabilitation and of physical activity after stroke, this trial will present a significant advance in clinical knowledge about physical fitness intervention after stroke.

This trial will make several unique contributions to the practice of cardiovascular active fitness therapy after stroke. Firstly, we will answer the question whether physical fitness intervention starting in the early rehabilitative phase after stroke will benefit their daily living - in particular in terms of gait speed and the BI. Secondly, we will provide first-time ever information on the effects of physical fitness intervention on language and cognitive function after stroke, and add further evidence to its effect on quality of life measures. Thirdly, we will help define an optimal physical fitness intervention prescription for people early after stroke. Given the wide availability of treadmill devices in rehabilitation settings across the world, the intervention would be transferable to inpatient (for example, Germany, Switzerland, Austria) and outpatient (for example, UK, USA) rehabilitation settings in the future. Fourthly, we will provide unique information about the mechanisms underlying physical fitness intervention, including laboratory markers of inflammation and the insulin-glucose pathway as well as markers of cerebral vasculature as well as functional and structural networks.

### Trial status

Patient recruitment will start in October 2013 and is aimed to continue for 30 months in total. Last follow-up is scheduled for October 2016.

## Abbreviations

ADL: activities of daily living; ANCOVA: analysis of covariance; BI: Barthel Index; FAC: functional ambulation category; PHYS: physical activity; PHYS-STROKE: Physical fitness training in Subacute Stroke; RELAX: relaxation

## Competing interests

The authors declare that they have no competing interests.

## Authors' contributions

AF led the conceptualisation, design, and implementation of this research protocol, and was the primary author for this manuscript. CW was a leader in the conceptualisation, design, and implementation of this research protocol, and is a contributing author for this manuscript. UG led the development of the statistical analysis plan. SH, MJ, JK, MS, EST, MG, CD and WF participated in the design of the protocol for interventions and assessments. RS participated in the implementation of the protocol. AHN participated in the implementation of the protocol and the setup for the laboratory analyses. AM participated in the design of the protocol. ME participated in the design and implementation of the protocol and the setup for the laboratory analyses. IW led the development of the data management protocol, and is a contributing author for this manuscript. All authors read and approved the final manuscript.

## Supplementary Material

Additional file 1 Description and rationale for secondary outcome measures, data management and quality control procedures, and study organisation and management. Click here for file

Additional file 2 List of participating institutions, principal local investigators and ethical body responsible for the respective centre. Click here for file
