# Supplementary material for: Physical fitness training in Subacute Stroke (PHYS-STROKE) - study protocol for a randomised controlled trial
Source: Trials. 2014 Feb 3;15:45. doi: 10.1186/1745-6215-15-45 (PMC3922602; doi:10.1186/1745-6215-15-45)
Supplement: Additional file 1 — Description and rationale for secondary outcome measures, data management and quality control procedures, and study organisation and management. [file 1745-6215-15-45-S1.doc]

***Additional file 1***

**Description and rationale for secondary outcome measures**

*Mobility (Gait endurance, Actigraph, Rivermead Mobility Index):*

In addition to walking speed, the distance a person can walk and the amount of daily walking that a person is able and willing to do are strong indicators of his or her health and condition . Thus, PHYS-STROKE also obtains a gait endurance test and monitors daily ambulation using an Actigraph. For gait endurance, the patients will walk for 6 min without interruption, and the maximum distance will be noted . If the patient has to stop during the 6-min test because of fatigue, the distance covered up to that point will be measured. Stumbling will not be considered a cause for stopping the test. To measure the amount of self-selected walking over an extended period of time, an actigraph will be used. The device is safe, highly accurate, unobtrusive for the wearer, capable of continuously recording data in short time increments, capable of withstanding daily use, with a 99% accuracy for recording steps with a variety of gait patterns . The device has also been successfully tested in monitoring ambulatory activity in persons with mild to moderate impairments post-stroke . Rivermead Mobility Index is a measure of disability which concentrates on body mobility, comprising a series of 14 questions and one direct observation, and covers a range of activities from turning over in bed to running .It is reliable to a limit of 2 points (out of 15), and its validity as a measure of mobility after stroke has been established.

*Motor Function*

Rivermead Arm Test is a subscale “Upper Limb/Extremity ('Arm') of the Rivermead Motor Assessment that determines motor performance of patients after stroke, and consists of 15 arm movements such as pronating/supinating the forearm, bouncing a ball, and functional items such as cutting putty, grasping and releasing objects, and tying a bow. It consists of test items in three sections that are ordered hierarchically, i.e. the first items are easier and become increasingly more difficult toward the end of the evaluation. The Box and Block test assesses unilateral gross manual dexterity to determine functional levels of the upper extremity in people with disability compared with those without disability . The Medical Research Council (MRC) scale is the accepted clinical tool for assessing muscle power . It is an ordinal scale that has shown high intra- and inter-rater reliability.

The resistance to passive movement (REPAS) scale is based on the Ashworth and the modified Ashworth scale , the most commonly used measures for spasticity/resistance to passive movement. The REPAS provides standards for both test administration and scoring of various passive limb motions, showing high internal consistency and reliability for the clinical assessment of resistance to passive movement in patients with central paresis.

*Cognitive function*

Cognitive function will be assessed using the Montreal Cognitive Assessment (MOCA), a brief screening tool for cognitive impairments that assesses multiple domains of cognitive functioning including short-term memory, visuospatial, executive and attentional function, concentration, working memory, language, and orientation to time and place . The test is available at www.mocatest.org and takes 10 min to administer Moreover, Trail Making Test A and B assessing cognitive speed, cognitive flexibility and visual search, as well as Category and Letter Fluency will be used as additional cognitive measures in all patients.

*Scales for disability, quality of life, sleep, mood*

The *modified Rankin Scale (mRS)* measures the degree of disability or dependence in the daily activities after stroke on a scale from 0-6, running from perfect health without symptoms to [death](http://en.wikipedia.org/wiki/Death). The mRS 3 months after stroke onset or later is recommended as an outcome measure in acute stroke trials .

The EuroQol 5 Dimension scale (EQ-5D) is a measure of self-reported quality of life and a health outcome that is applicable to a wide range of health conditions and treatments and high reliability and validity. It describes health in terms of five dimensions: Mobility, Self-care, Usual activities, Pain/discomfort, and Anxiety/depression. The EQ-5D-5L version was developed by the EuroQol Task Force in 2005 to increase sensitivity and to reduce ceiling effects of previous versions , by using five levels for each dimension, i.e. no problems, slight problems, moderate problems, severe problems, and extreme problems.

The Pittsburgh Sleep Quality Index (PSQI) is a self-rated questionnaire which assesses sleep quality and disturbances over a 1-month time interval . Nineteen individual items generate seven "component" scores: subjective sleep quality, sleep latency, sleep duration, habitual sleep efficiency, sleep disturbances, use of sleeping medication, and daytime dysfunction. The sum of scores for these seven components yields one global score.

The Center for Epidemiological Studies Depression (CES-D) scale , a 20-item self-report scale that assesses primarily non-vegetative symptoms of depression (sum of items rated from 0 [rarely or none of the time] to 3 [most or all of the time] with a maximum score of 60, α = 0.95; sample items: “I felt depressed” and “I talked less than usual”).

*VO2 max and Gait Energy Expenditure*

In order to assess the VO2 max and Gait Energy Expenditure [VO2·s-1=(ml of O2 min-1 · kg-1)/(m · min-1)], oxygen consumption (VO2), carbon dioxide production (VCO2), pulmonary ventilation (VE) and heart rate (beats/minute) will be assessed in each subject at each measurement point with the help of a telemetric apparatus (K4b2, Cosmed, Italy). This is a lightweight, portable system widely used and standardized for assessment of these parameters that consists of a facemask for collecting expired air, sensors for analyzing oxygen and carbon dioxide content of expired air, a heart rate monitor, a battery pack, and a transmitter worn by the subject. A receiving unit will receive and store the transmitted data. The gas analyzers will be calibrated with room air and a sample of known gas, and the flowmeter will be calibrated by moving a known volume of air through the flowmeter with a calibration syringe according to the manufacturer’s instructions. The machine will be calibrated before testing each subject. Initially, the patients will be familiarized with the set-up at rest. Then the patient will perform the six minute walking test (see section above for details). The values will be recorded on-line and transmitted to a computer. The overall walking energy cost (WEC) will be calculated by dividing oxygen consumption by gait speed and converted to J · m-1 · kg-1,  assuming that 1ml O2 consumed in the human body yields 20.9J (which is strictly true only if the respiratory quotient equals 0.96). The overall walking cardiac cost (WCC) will be calculated as the number heart beats per unit distance (beats · m-1), by dividing heart rate by gait speed (for more details see ).

*Functional Ambulation Classification*

The Functional Ambulation Category (FAC) is a reliable and valid score to help assess gait ability. Standardized definitions are used to classify the participant according to one of six categories (0-5) during a 6 minute walk, giving detail on the physical support needed by patients while walking, irrespective of the technical aids used. Level 0 indicates a patient who cannot walk at all or needs the help of two therapists. Level 5 indicates a patient who can walk everywhere, including stairs, independently. The FAC will be recorded at each study assessor visit, as well as before each intervention.

*Cardiovascular risk factors including blood pressure, markers of inflammation, the insulin-glucose pathway, lipid profile, and others*

Resting systolic and diastolic blood pressure as well as heart rate will be recorded at the beginning of the assessment along with weight, and waist-to-hip ratio. Moreover, markers of inflammation and the insulin –glucose pathway, lipid profile, hormones, hemogram, liver and kidney function, and coagulation parameters will be determined from a peripheral blood draw. These parameters are known to significantly influence vasculature and stroke risk and may be amenable to physical fitness intervention . In order to investigate the influence of physical activity on the recovery from stroke-induced immunodepression, we will aim to identify and characterise markers of peripheral immunity such as monocytic HLA-DR expression. In addition, we will search for gene expression signatures using transcriptome analysis from peripheral blood leukocytes. The ultimate aim of these analyses is to predict long-term outcome after stroke .

Moreover, to retrospectively assess integrated cortisol secretion over approximately three months prior to inclusion into the trial, hair cortisol concentrations (HCC) will be determined. Here, a 3-cm hair segment will be taken from each participant at baseline. Since cortisol is being incorporated into the growing hair, HCC are assumed to provide a retrospective picture of integrated cortisol secretion over periods of several months .

# Data Management and Quality Control Procedures

Paper-based case report form (CRF) documentation will be used. After monitoring, the original CRF pages will be sent to data management; the copy remains in the center. To ensure data quality, a good clinical practice (GCP) monitoring will be held on a regular basis.

All data will be collected after monitoring by a relational data base. The PHYS-STROKE database system facilitates data entry with built in quality control checks (e.g., range checks, checking for missing data for required data points).Verification of accuracy will also be done by range, validity and consistency checks. Implausible or missing information can be corrected or supplemented in consultation with the study physician and / or the study assessor. The adjustment documents will be kept with the case report forms. The validated data will be stored in a database.

In addition, a PHYS-STROKE web-based system provides a public website including study information, news items, employment opportunities, publications.

Additional quality control procedures include: (1) randomly selected comparison of data points between the paper records and the electronic records in the SQL database at each study site, and (2) frequency distributions checks of key outcome variables (overall, not stratified by treatment assignment) along with a list of participant IDs associated with possible outliers or questionable data points.

# Study Organization and Management

The PHYS-STROKE Trial is managed by an executive steering committee. This committee is responsible for the overall trial management including oversight of participant recruitment, execution of the trial, ethical conduct of the trial, study publications and ancillary studies. The steering committee includes the Principal Investigator Dr. Agnes Flöel, the Co-principal Investigators Dr. Cordula Werner and Dr. Martin Ebinger, and the neurorehabilitation consultants of the respective centers. Dr. Ian Wellwood, leads Clinical Epidemiology and Health Services Research Group (CEHRiS) that oversees data management and analysis with Dr. Ulrike Grittner as the study lead statistician. Moreover, Moreover, a patient representative is invited and contributes to meetings of the executive steering committee.

Dr. Flöel, the study principal investigator chairs the committee. The executive steering committee has conference calls every month and meets once a year in person. Each rehabilitation intervention site receives oversight from a clinical research coordinator. The Clinical research coordinator has a weekly conference call with the site responsible physician. The clinical research coordinator, the study assessor and the site physicians have monthly conference calls to ensure that all assessment, intervention, and patient recruitment issues are dealt with in an efficient and consistent manner across sites.

There is a CSB appointed DSMB to oversee the trial. The DSMB is responsible for assuring that the study is safe and conducted according to high scientific and ethical standards. The DSMB assesses participant recruitment, retention and follow-up, and data quality. The DSMB also reviews all adverse events and monitors safety issues. It reviews all proposed protocol changes and all ancillary study proposals.

Then DSMB meets with Dr. Flöel, Dr. Wellwood, Dr. Grittner, and a CSB board member after each formal evaluation (after 50 patients). A summary of the DSMB meeting and subsequent recommendations are forwarded to the Executive committee.

**References**
