# Supplementary material for: Physical fitness training in Subacute Stroke (PHYS-STROKE) - study protocol for a randomised controlled trial
Source: Trials. 2014 Feb 3;15:45. doi: 10.1186/1745-6215-15-45 (PMC3922602; doi:10.1186/1745-6215-15-45)
Supplement: Additional file 2 — List of participating institutions, principal local investigators and ethical body responsible for the respective centre. [file 1745-6215-15-45-S2.doc]

Additional file 2

**List of participating institutions, principal local investigators and ethical body responsible for the respective centre**

| **REHABILITATION CENTRE** | **PRINCIPAL INVESTIGATOR** | **ETHICAL BODY** |
| --- | --- | --- |
| Brandenburg Klinik, Bernau-Waldfrieden | Michael Jöbges | Landesärztekammer Brandenburg |
| Evangelisches Geriatriezentrum Berlin | Elisabeth Steinhagen-Thiessen | Charité Universitätsmedizin Berlin |
| Klinik Beelitz, Beelitz-Heilstätten | Wolfgang Fischer | Landesärztekammer Brandenburg |
| MEDIAN Klinik Grünheide | Janet Knauß, Michael Seifert | Landesärztekammer Brandenburg |
| MEDIAN Klinik Berlin-Kladow, Berlin | Christian Dohle | Charité Universitätsmedizin Berlin |
| Medical Park Berlin Humboldtmühle, Berlin | Stefan Hesse | Charité Universitätsmedizin Berlin |
